# Difference in evolutionary patterns of strongly or weakly selected characters among ant populations

**DOI:** 10.1038/srep39451

**Published:** 2016-12-20

**Authors:** Shuichiro Imai, Kazuya Kobayashi, Yusaku Ohkubo, Norihiro Yagi, Eisuke Hasegawa

**Affiliations:** 1 Laboratory of Animal Ecology, Department of Ecology and Systematics, Graduate School of Agriculture, Hokkaido University, Kita-ku, Sapporo 060-8589, Japan

## Abstract

Despite being a central issue in evolutionary biology, few studies have examined the stasis of characters in populations with no gene flow. A possible mechanism of such stasis is stabilizing selection with similar peaks in each population. This study examined the evolutionary patterns of morphological characters with and without strong selection in ant populations. We show that compared to a character that seems to be less important, characters that are more important were less variable within and among populations. Microsatellite analyses showed significant genetic differences between populations, implying limited gene flow between them. The observed levels of genetic differentiation cannot be attributed to recent population separations. Thus, the observed differences in morphological variance seem to reflect the degree of selection on each character. The less important character changed proportionately with time, but such a pattern was not observed in more important characters. These results suggest that stabilizing selection maintains morphological stasis between populations of the same species with minimal gene flow independent of divergence times.

Understanding the forces responsible for generating biodiversity is one of the most important goals of evolutionary biology. Consequently, the mechanisms underlying diversification among populations have attracted considerable attention^1^,^2^,^3^,^4^,^5^,^6^,^7^,^8^,^9^,^10^,^11^. While gene exchange above a certain threshold is considered to prevent populations from differentiating^12^,^13^,^14^, local adaptations can result in differentiation between populations with minimal gene flow^10^,^15^,^16^,^17^,^18^,^19^.

Among populations with minimal gene flow, a common stabilizing selection pressure can result in stasis in the status of a character^20^. As a result, an evolutionary pattern in which the amount of character change does not correlate with time is expected. In contrast, characters under no (or weak) selection are expected to show time-dependent changes because of the effects of random genetic drift^21^. Although molecular evolution supports these views based on biased ratios between synonymous and non-synonymous nucleotide substitutions^22^, examinations of morphological characteristics are needed to confirm the existence of these mechanisms in relation to evolution of morphology. Comparing evolutionary patterns for morphological characters with and without selection is thus important for understanding the mechanisms generating diversity and similarity among populations within a species. There have been a few studies focused on the evolutionary mechanisms responsible for promoting stasis among populations^20^,^23^, but no study has compared evolutionary patterns between characters with and without selection.

In this study, we compared the evolutionary patterns of characters under strong or weak selection among ant populations with minimal gene flow. For each character with a different functional importance, we investigated its degree of variation within and among populations. Then, the amount of evolutionary change, along with the phylogenetic branching pattern, was compared with branch lengths using a phylogenetic tree reconstructed from genetic distances among populations, which were estimated from 4 microsatellite loci. Based on the results, we discuss the factors affecting the evolutionary patterns for characters experiencing different strengths of selection.

## Results

### Function and frequency of use in the examined characters

We compared the frequency of use of two morphological structures (tibial spur of the right front leg, FTS; and tibial spur of the right hind leg, HTS) by using 10 *Formica yessensis* workers collected from a population in Ishikari (see Supplementary Fig. S1). During the observations, all the workers frequently used their FTS to clean their antennae, while they seldom used their HTS, which was only observed for use in scratching the back surface of the abdomen where no apecial organs are found. The frequency of use of each character during an hour of observation was 371.9 ± 59.0 (mean ± S.D., n = 10) and 0.20 ± 0.40 for the FTS and the HTS, respectively. The difference was highly significant (t-test, t = 19.931, df = 18, p < 0.0001), suggesting that the FTS experiences stronger selection than the HTS.

One may think that the frequency of use is not useful for estimating the adaptive significance of organs because, for example, insects’ copulatory organs are adaptively important but are used only a few times in their life. Thus, we provide an additional explanation for the logical basis of our assumptions (strong selection on FTS length (FTSL) and weak selection on HTS length (HTSL)) in detail. First, worker ants from all the populations examined used their FTS for cleaning their antennae. The antennae of ant workers are their most important sensory organ, and many chemical sensory receptors exist on its surface. If a worker ant goes without cleaning her antennae for long time, she loses her most important sense (ants are not highly dependent on vision). Thus, FTSL in all populations must be optimized for efficiently cleaning antennae. In fact, the shape of the FTS is quite different from that of other homologous traits (MTS and HTS). The FTS has a brush-like shape to clean the surface of antennae (see Fig. S2), but the MTS and HTS are simple spines. These facts indicate that the FTS is a special organ used to clean antennae and that it is under a strong stabilizing selection to maintain this function. This provides a strong basis for assessing selection on FTSL independent from the frequency of FTS use. Second, we personally observed many ants from multiple populations during much longer time periods than reported above and never observed uses for the HTS other than the reported behaviour (infrequently used to scratch the back surface of the abdomen). In addition, anatomically, the HTS is not likely used to manage any important organ of ants. Thus, these facts support the assumption of weak selection on this character irrespective of its frequency of use. Thus, we assumed strong selection on the FTS and weak selection on the HTS, and selected these characters for this study.

In addition, on September 6, 2016, we collected 50 workers from the shore in Ishikari and calculated the residuals for FTSL, HTSL and the length of the tibial spur of the right middle leg (MTSL) from an allometric equation with head width. Differences in the residual variances were compared for each pair of the three characters using *F* tests. After a Bonferroni correction for multiple comparisons, the residual variance of FTSL was found to be significantly smaller than that of MTSL and HTSL. Whereas, there was no difference between MTSL and HTSL (for detail, see Supplementary Table S2). These results suggest weak (or no) stabilizing selection on MTSL and HTSL and strong stabilizing selection on FTSL. Note that we did not observe the use of the MTS by any worker for any purpose.

Another measured character, the length of tibia of the right hind leg (HTL), could also experience strong selection based on the fact that the walking speed of a worker was reduced to approximately 48% after cutting the HTL at an intermediate point (for experimental details and results, see Supplementary Fig. S3). This result clearly shows that a change in HTL results in a serious reduction of individual performance. Similarly, any mutation that brought about such an inconvenient change will be quickly selected out of the population. Thus, it is reasonable to assume the presence of strong stabilizing selection on HTL.

### Fragility of species identity and data combination

**Figure 1:**
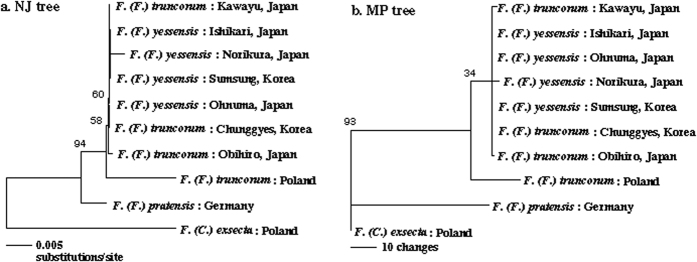
Phylogenetic relationships among the populations examined inferred from COI sequences. (**a**) and (**b**) are the NJ tree and one of the three MP trees, respectively. The numbers above the nodes represent bootstrap probabilities greater than 30%.

We examined 10 populations of *F. yessensis* and *F. truncorum* from Japan and Korea (referred as far-east populations; see Supplementary Fig. S1). First, we performed a statistical test to assess species identity for both species to determine if data could be combination for these far-east populations. If far-east populations of *F. yessensis* and *F. truncorum* have genetically differentiated from each other in substantial manner, a given species distance between two populations (1 or 0, belonging to the same species or not) will be correlated with the genetic distance irrespective of the geographic distance. The partial correlation analysis showed no significant correlation between the species distance and the genetic distance when the geographic distance was controlled for (Partial Mantel test, Kendall’s τ = 0.105, p = 0.178). This result indicates that the present taxonomic status is not robust (see also Fig. 1), suggesting that we can combine data for the far-east populations.

A COI gene sequence of 1041 nt (GenBank Accession No., AB103355-AB103364) was used to reconstruct a molecular phylogeny. All three methods (Bayesian inference, maximum parsimony and neighbour joining) resulted in a branching pattern in which the Far-East (Japan + Korea) populations were derived from European *F. truncorum* (Fig. 1). While there were insufficient synapomorphic substitutions to resolve the phylogenetic relationships within the far-east populations, this result suggests that these populations have undergone diversification from a common ancestor (presumably a common ancestor shared with European *F. truncorum*) after arriving at this area. A combination of the data for *F. truncorum* and *F. yessensis* in the far-east populations is thus further justified.

### Morphological differences among populations

Head width (HW) was used as an index of body size as this character was found to be the most isometric for PC1 in an analysis including all the four characters^24^. The allometric relationships of the lengths of the tibial spurs on the right legs (right fore leg spur length, FTSL; right hind leg spur length, HTSL) relative to HW were then calculated (Supplementary Table S1). An ANCOVA showed that the slopes of all the characters significantly varied among the populations (see Supplementary Table S1), indicating that each character has significantly diversified among the populations.

The variance associated with the slopes of HTL, FTSL and HTSL among the populations was 0.90 × 10^−5^, 1.86 × 10^−5^ and 11.17 × 10^−5^, respectively. HTSL exhibited a considerably larger variance than the other two characters among the populations. Simple comparisons revealed significant differences in the degree of variance between FTSL and HTSL (*F* test, *F* = 6.658, df = 7, 7, P = 0.036) and between HTL and HTSL (*F* test, *F* = 21.655, df = 7, 7, P = 0.0016), but the difference between HTL and FTSL was not significant (*F* test, *F* = 3.2523, df = 7, 7, P = 0.09). These results show that although HTL and FTSL were significantly different among the populations (see Supplementary Table S1), HTSL was the most variable character within and among the populations. In addition, the above results support our hypothesis that HTL and FTSL are under strong stabilizing selection pressures, but HTSL is not.

No significant correlation was detected between the slopes obtained and the latitude of the collection site (HTL: r = −0.259, P = 0.554; FTSL: r = −0.233, P = 0.595; HTSL: r = 0.072, P = 0.872). Thus, a geographic cline was not detected for any character.

### Residual variances within populations

The residuals from an allometric regression of a character relative to HW were calculated for each character in each population, and the variance in the residuals (residual variance) was calculated for each population (Table 1). In all the populations, the variance in the residuals relative to the allometric regression line was the largest for HTSL, intermediate for FTSL and the smallest for HTL (Table 1), and the differences were statistically significant between all combinations of the characters (paired *t*-test, df = 7: HTL vs. FTSL, *t* = 4.379, P = 0.0032; HTL vs. HTLS, *t* = 4.884, P = 0.0018; FTSL vs. HTSL, *t* = 4.504, P = 0.0028). These results suggest that although HTL shows the strictest allometric constraints, FTSL is more strictly constrained than HTSL. As a result, the ranking of characters in relation to the strength of selection pressure was estimated to be as follows: HTL > FTSL > HTSL.

There are variances in the strength of selection on HTSL among the populations because HTSL significantly varies among the populations (see Supplementary Table S1). In fact, several populations show allometric slopes steeper than unity for HTSL. (slope (S) > 1; Kawayu, Obihiro, Ishikari, Ohnuma, Norikura). In these populations, the average residual variance of HTSL is 550.2 ± 30.8 (mean ± S. D.) and that of FTSL is 95.1 ± 7.8, whereas in the other populations (S < 1; Furano, Moshiri, Gotemba), the former is 112.9 ± 11.2 and the latter is 41.2 ± 4.6. Because the average residual variance in HTSL in the former populations (S > 1) is significantly larger than that of the later populations (S < 1) (*t*-test, *t* = 2.131, df = 4, p = 0.0385), there may be differences in the strength of selection on HTSL among populations (selection is stronger in the populations with S < 1). However, even in the populations with S < 1, the average residual variance in HTSL is marginally larger than that of FTSL (*t*-test, *t* = 2.92, df = 2, p = 0.054). This marginality difference could be due to the small sample size (n = 3). These results support the following view: although there are differences in the strength of selection on HTSL among populations, the selection strength is consistently stronger on FTSL than on HTSL in each population.

Another possibility is that the HTS is used as a weapon because insect weapons are selectively important but frequently show a large variance within populations, such as in the stag beetles, which shows dimorphism in its mandible^25^. However, we never observed the current ants using their HTS as weapons. In the dimorphic male stag-beetle, *Prosopocoilus inclinatus*, the lengths of their mandibles (weapons) show a strong allometric constraint within each morph, i.e., the residual variance is small within each morph^25^. Furthermore, in the current ants, all the allometric regression lines for HTSL on HW within each population show linear trends without any inflection point, indicating monomorphism for HTSL from the viewpoint of allometry. Thus, the large residual variance in HTSL is not attributed to HTSL polymorphism. As a result, weak stabilizing selection on HTSL remains a likely explanation of the large residual variances for this character within and among the populations.

### Population genetics based on the microsatellites

We determined the genotypes of a total of 911 workers from the 10 populations for 4 microsatellite loci. Significant linkage disequilibrium was not detected for any pair of loci, indicating that every locus is independent of each other. Inbreeding coefficients (*F*_IS_) were not significantly positive in 9 of the 10 populations, with the exception of the values for the Furano population (Furano: *F*_IS_ = 0.158, P < 0.0001), indicating that inbreeding does not occur in the most populations. The average relatedness between workers in a nest was not significantly different from zero in 7 of the 10 populations, with the Ishikari, Furano and Chunggyesan populations being the exceptions. However, even in these populations, the estimated values were close to zero (mean ± 95% confidential limit: Ishikari, −0.022 ± 0.018; Furano, 0.0091 ± 0.0086; Chunggyesan, 0.262 ± 0.226), suggesting that multiple workers from a nest reflect each queen’s genotype.

### Genetic differentiation and phylogenetic relationships among populations

**Figure 2:**
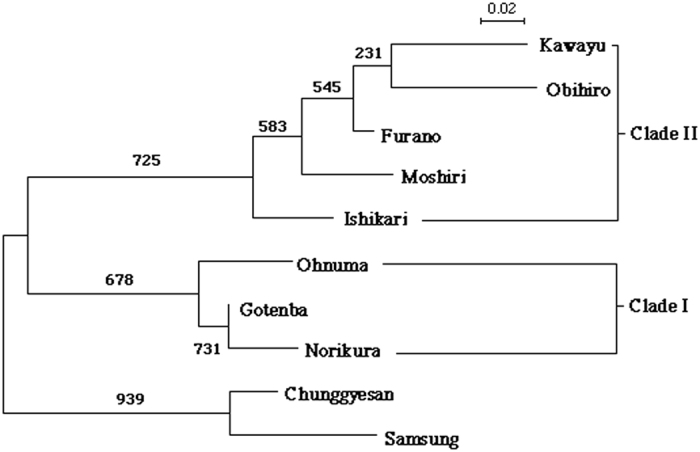
Phylogenetic relationship among Far-East *Formica* spp. populations. Phylogeny was estimated by-locus bootstrap using the chord distance (Dc), from allele frequencies at four microsatellite loci. The numbers above the nodes represent bootstrap probabilities from 1000 replicates.

The fixation index (*F*_*ST*_) among the populations ranged from 0.023 to 0.488. All except one of the values were significantly different from zero after a Bonferroni correction for multiple comparisons with a strict significant level of p = 0.001 (Permutation test for all pairs with one excluded, p < 0.00001; significance level after Bonferroni correction, p = 0.0005). In the Gotemba-Norikura pair, the *F*_*ST*_ scores did not significantly differ from zero after the Bonferroni correction (*F*_*ST*_ = 0.023, Permutation test, p = 0.009; significance level after Bonferroni correction, p = 0.0005). Figure 2 shows the NJ tree based on the chord distance (Dc) for the Far-East populations by locus bootstrap values from 1000 replicates. The Japanese populations formed two major clades, i.e., Norikura + Gotemba + Ohnuma (Clade I) and the other populations from Hokkaido (Clade II). There was a large gap in the Dc between the two clades. The Korean populations were closer to the members of Clade I. This distance-based phylogeny also suggests the fragility of the present taxonomy of these species because several *F. yessensis* populations (Ishikari and Samsung) formed a robust clade with *F. truncorum* (Fig. 2). This provides additional justification for combining the data from these species, and thus, we used the combined data in the following analyses.

### Ancestral states and evolutionary patterns of the characters

We reconstructed the ancestral states of each of the three characters (HTL, FTSL and HTSL) at each branching point within the above tree using the Kutsukake and Innan’s (KI) method^26^ (see the Methods section for details). The independent contrast analysis revealed no significant correlation between any pair of characters (FTSL vs. HTSL: r = −0.090, z = −0.181, df = 7, p = 0.8561; HTL vs. FTSL: r = 0.595, z = 1.370, df = 7, p = 0.1708; HTL vs. HTSL: r = −0.044, z = −0.087, df = 7, p = 0.9303), suggesting that these characters have evolved independently from each other. Moreover, the strengths of stabilizing selection, represented by the variable α in the KI method, for HTL, FTSL and HTSL were estimated as (mean ± SD) 0.770 ± 0.163, 0.698 ± 0.213 and 0.335 ± 0.203, respectively. These estimated values were significantly different from each other (one-tailed *t*-test; HTL vs. FTSL: *t* = 2.7113, df = 183, p = 0.0037; HTL vs. HTSL: *t* = 16.566, df = 188, p < 0.0001; FTSL vs. HTSL: *t* = 12.164, df = 195, p < 0.0001). Thus, the order of characters in relation to stabilizing selection strength was again estimated to be as follows: HTL > FTSL > HTSL.

Next, we examined whether the amount of evolutionary change in each character is correlated with branch lengths. Because there is a large gap in the distance between Clade I and II, we conducted the analysis on each the clade. This treatment is required because the ancestor of Clade I is assumed to have come to Japan through the Korean peninsula, whereas that of Clade II probably arrived in Japan via Sakhalin. No characters showed a correlation with branch length within Clade I (for all characters, p > 0.05). A significant positive correlation was detected for HTSL in Clade II (r = 0.724, z = 2.049, df = 8, p = 0.0405), but HTL and FTSL did not show such a trend (HTL: r = 0.548, z = 1.376, df = 8, p = 0.169; FTSL: r = 0.602, z = 1.558, df = 8, p = 0.1193). These results show that only HTSL in Clade II has evolved in a manner corresponding to the time after branching.

## Discussion

The independent contrast analysis did not reveal any evolutionary correlation among the characters, implying that the three characters evolved independently from each other. Thus, we can interpret the observed evolutionary pattern of each character independently. We found that the magnitude of the residual variance within a population corresponded to the degree of divergence among the populations (see Table 1 and the Results section). The degree of variation is the largest for HTSL, intermediate for FTSL and the smallest for HTL, both within and between populations (Table 1, Supplementary Table S1). While the FTS is used to frequently clean antennae, which the most important sensory organ for an ant, the ants seldom used their HTS and only for a seemingly less important purpose. HTL is likely under a strong stabilizing selection pressure, as supported by the fact that the walking performance of worker ants was reduced by approximately 48% after cutting off the right hind tibia at the mid-point (see Supplemental Figure S3). Thus, it is reasonable to assume that these three characters are each subjected to different strengths of stabilizing selection, and the results suggest that the order of the characters in relation to the strength of selection is HTL > FTSL > HTSL. The results show that HTL and FTSL are more stable than HTSL, both within and between populations. Because a previous study^10^ has shown the existence of a genetic basis in allometric patterns, the observed variations in the residual variance seem to reflect differences in the strength of natural selection on the allometric pattern associated with each character.

Few studies have focused on the evolutionary forces responsible for maintaining stasis within a species between distant populations^20^. A model of morphological stasis based on stabilizing selection has been presented that shows a good fit with the observed pattern of phenotypic divergence data over a variety of timescales^27^. In our study, the obviously functional HTL and FTSL were more stable among populations than the seemingly less functional HTSL. The microsatellite analysis showed that significant genetic differentiation had occurred among the populations surveyed, except for a pair of populations (i.e., Gotemba and Norikura). If gene flow between populations is sufficient, the frequencies of neutral alleles do not differ between populations^8^. Thus, our results indicate minimal gene flow between populations.

A shortage of time after population separation is another factor that could explain minimal genetic differentiation between populations. However, significant genetic differentiations were observed in this study, suggesting that enough time has passed for the accumulation of genetic mutations after the separation of each population. We therefore cannot attribute the observed stasis in HTL and FTSL to either sufficient gene flow between populations or a shortage of time after population separation. It therefore remains likely that a narrow range in the stabilizing selection peaks maintains HTL and FTSL at similar states between genetically diverse populations. This view is also supported by the facts that the variances among populations in the allometric slopes of these two characters were smaller than that of HTSL, suggesting that the stabilizing selection peaks for HTL and FTSL were similar among the populations.

There is a large gap in the Dc between the southern (Clade I) and northern populations (Clade II; Fig. 2), with an apparent division existing between the Ohnuma and Ishikari populations, although both of the populations are in Hokkaido. Interestingly, several studies have reported the existence of a biogeographical border between Ohnuma and Ishikari for a wide range of taxa, e.g., plants^28^, butterflies and moths^29^, ground beetles^30^ and ants^31^. In addition, the old Japanese Archipelago was once connected to both the Korean peninsula and Siberia, enclosing the old Sea of Japan^32^,^33^,^34^. It is therefore possible that Clades I and II are derived from the Korean peninsula and from Siberia via Sakhalin, respectively. Indeed, the Korean lineage joined to the tree at the internode between Clades I and II (Fig. 2). Although a comparison including Sakhalin populations is required to confirm the above scenario, the obtained results do not contradict to this view.

Clade I contains too few OTUs (n = 3) to allow for a robust statistical analysis. In addition, if the ancestral population of Clade I arrived in Japan through the Korean peninsula and that of Clade II came to Japan from Sakhalin, it is not appropriate to analyse the data by combining both clades. When only Clade II was analysed, a positive correlation was observed between the degree of change along a branch and branch length for HTSL but not for HTL and FTSL, suggesting that only HTSL has evolved in proportion to the divergence time. As discussed in the previous section, stabilizing selection with similar optimal peaks might act on HTL and FTSL. The narrow ranges of the optimal peaks among the populations would permit only small changes along a phylogenetic branch regardless of the branch length. Our results support the hypothesis that morphological similarities between far distant populations within a species (without gene flow) are maintained by similar stabilizing selection pressures in each population. When a species extends its distribution to a new area, they are likely to inhabit an area with a suitable ecological niche. In such a place, stabilizing selection pressures similar to those of their previous habitat are likely to act on the species. In other words, individuals of a species are more likely to survive in new habitats where selection pressures similar to those of their previous habitat are present as they are already adapted to such an environment. Thus, stabilizing selection would preserve similar morphologies among far distant populations in a species without gene flow, as is shown by the present study. More studies using more characters are required to determine the robustness of the above hypothesis. In particular, MTSL is a good subject for testing this hypothesis because MTSL shows similar degrees of residual variance to those found in the allometric relationship for HTSL, suggesting weak (or no) stabilizing selection on this character. Whether MTSL shows a pattern of evolution that correlates with time as indicated by branch lengths on the phylogenetic tree is worth testing in the future.

In the absence of selective pressures, allele frequencies in a population change in relation to divergence time via random genetic drift^21^. The large inter-population variance in HTSL might result from differences in the direction of selection among the populations. However, in such a case, small variances would be expected within populations, but the results obtained appear to contradict such an outcome. Another possibility is that the HTS is a specific trait, similar to the weapons of horned beetles mentioned above. However, if this is the case, evolutionary changes would not show a time-dependent pattern. All of the results obtained for HTSL (seemingly less functional importance, large residual variances within and among the populations, and the time-dependent manner of evolutionary change) support the idea that HTSL has been affected by random genetic drift. Additional empirical investigations on the evolutionary forces that generate diversity and similarity among populations need to be undertaken.

## Methods

### Study organism

The ants used in this study, *Formica truncorum* (Fabricius) and *F. yessensis* (Forel), have huge polygynous colonies consisting of numerous adjacent nests that are shared by all colony members^35^. New queens mate with males on the ground without nuptial flights and then return to their natal nests^36^. Thus, population density is expected to be high^37^,^38^. In Japan, red wood ants inhabit high elevations, and most populations are geographically isolated. Thus, gene flow between populations is expected to be minimal. Taken together, these characteristics imply that these ants are well suited for use in the study of character evolution.

### Species identification

*F. yessensis* is found in southwestern Hokkaido and the highlands of Honshu in Japan, on the Korean peninsula and in Taiwan, while the range of *F. truncorum* extends from northeastern Hokkaido to Europe^39^. The criterion used for the identification of these species was the number of hairs on the hind tibia^39^. However, in Hokkaido, the existence of a geographical cline was observed among populations for this character (F. Ito, pers. com.). Thus, the current classification seems to be fragile. If each *F. yessensis* and *F. truncorum* population is reproductively well-isolated, we should detect a clear genetic differentiation between the two species when the geographic distances among populations is controlled for. This hypothesis was tested statistically using a partial correlation analysis. We assigned a pseudo-species distance to each population pair (0 to pairs of the same pair and 1 to pairs of different species). Then, Kendall’s partial rank correlations between the species distances and genetic distances were calculated while controlling for geographic distances.

Using mitochondrial DNA sequences, we also conducted a phylogenetic analysis among the populations to test whether combining the data was possible (see the Results section for details). When no clear genetic differentiation was found between *F. truncorum* and *F. yessensis*, we considered it is reasonable to combine data for these two species for Far-East populations.

### Sample collection

Five populations of each species were collected in Far-East Asia (see Fig. S1 in Supplementary Information). Except for the Korean populations, more than 100 workers were collected from 10 nests at each locality from 1999 to 2002. We were unable to collect samples from 10 nests in Korea, as there were only a few nests at the collection sites. Approximately half of the ants collected from each nest were preserved in a 10-ml glass vial filled with 75% ethanol for measurements of morphological characters, while the remaining ants were preserved in acetone for DNA analyses. The workers of both species are continuously polymorphic and do not have morphologically distinct subcastes^40^, which implies that all workers follow a single allometric growth pattern. Considerable care was taken to collect ants belonging to all size ranges at each sampling site.

For additional experiments and measurements, we collected 50 workers of various sizes from the Ishikari population on September 6, 2016.

### Measurement of morphological characters

We measured the following four characters, the tibial spur (FTS) length of the right foreleg (FTSL), the tibial spur (HTS) length of the right hind leg (HTSL), the tibial length of the right hind leg (HTL) and head width (HW) (see Supplementary Fig. S2). For the additional samples, we also measured the tibial length of the right middle leg (MTSL) in addition to the above four characters. HW was used as an index of body size because this character was found to be the most isometric for PC1 in a principal component analysis using all the four characters (see ref 24. p203–205).

Preliminary observations using many ants from multiple populations showed that the FTS is used frequently by the ants to clean their antennae, while the MTS and HTS have no apparent function except for the HTS being rarely used to scratch the back surface of the abdomen, where no specific organ exists. We observed 10 *F. yessensis* workers collected from the Ishikari population for an hour and recorded the frequency of FTS and HTS use.

We measured the four characters (FTSL, HTSL, HTL and HW) of 30 workers from each of the 10 nests in each population. The head and right leg of each collected worker were removed from the body, and each character was measured to the nearest 0.001 mm using a binocular microscope (SZH-ILLD, Olympus, Tokyo, Japan) fitted with an ocular micrometer. The measurements were ln-transformed to allow for comparisons between characters with different mean variances^41^. For the additional samples, the removed heads and legs from each of the 50 workers collected were adhered to a glass slide and photographed with a scale (1 mm). The size of each character was measured to the nearest 0.001 mm using ImageJ ver. 1. 5. 1F software to analyse the photographs.

### Evaluation of morphological differences among populations

Since both the species are continuously polymorphic^40^, the mean values and variances of the measured characters could not be compared directly to evaluate morphological differences among the populations. Because the three characters (FTSL, HTSL and HTL) of interest are considered to exhibit an allometric growth pattern relative to body size, the slope of the allometric equation relative to HW can be used as an index that characterizes each population. The allometric slope was calculated for each colony in each population and was used to compare the populations. Furthermore, we assessed the correlation between the allometric slopes for each character and the latitudes of the collection sites to examine the relationship between the allometric relationships and geographic clines.

### Strength of allometric constraints for each character

To examine the strictness of the allometric constraints for the characters examined, we calculated the variances in the residuals relative to the allometric line for each character in each colony from each population. When a character has a strong allometric constraint, the residual variance is expected to be small and vice versa^41^,^42^,^43^. Thus, the magnitude of the residual variance was used as an index of the strength of selection.

### Preparation of DNA

We used two types of genetic information to generate phylogenies for the populations, mitochondrial DNA (mtDNA) sequences and allele frequencies of genomic microsatellite loci. A DNA extraction kit (DNeasy Tissue Kit, Qiagen) was used to prepare DNA for mtDNA sequencing. Genomic DNA for microsatellite analyses was prepared using a modified Chelex method^44^. A leg of each acetone-preserved specimen was placed in a 1.5-ml microcentrifuge tube along with liquid nitrogen and was crushed, and total DNA was extracted. In the Chelex extraction, the crushed leg was incubated in 250 μl of Chelex solution (10% Chelex resin (Bio-Rad) in Tris-EDTA (pH 8.0) buffer) containing 5 μl of Proteinase K solution (20 mg/ml) for 12 hrs at 55 °C. After the incubation, samples were boiled at 95 °C for 5 min to inactivate the proteinase.

### Analysis of mtDNA

We sequenced a section (1041 nt) of the cytochrome oxidase subunit I (COI) gene encoded on the mtDNA. The target region was amplified via PCR using the primer pair CI13 and CI24^45^ and was sequenced using a cycle sequencing kit (DMTS Quick Start Kit, Beckman and Coulter) with an automated sequencer (CEQ2000, Beckman and Coulter). The phylogeny was reconstructed using three methods, Bayesian inference (Bayes)^46^,^47^, maximum parsimony (MP) and neighbour-joining (NJ)^48^ using Mr. Bayes (ver. 3.2.1)^46^ or PAUP* (ver. 4.0b10)^49^. *Formica (Coptoformica) exsecta* from Finland and *Formica (s str.) pratensis* from Germany were used as far and near outgroups, respectively. *F. truncorum* from Finland was also included in the analysis to determine the relationship between Japanese *F. yessensis* and *F. truncorum* populations.

### Microsatellite analysis

We used four polymorphic microsatellite loci (Fy3, Fy4, Fy7 and Fy13) to characterize the genetic composition of the populations. The primer sequences and amplification conditions have been published elsewhere^50^. For microsatellite genotyping, we sampled approximately 10 workers from each nest. A total of 911 workers were genotyped at the four loci using polyacrylamide gel electrophoresis with silver staining^50^,^51^. We used the optimal number of PCR cycles for each locus^50^ to prevent incorrect genotyping due to the occurrence of ‘phantom’ alleles caused by too many PCR cycles^52^. To avoid misinterpreting the genotypes of the individuals examined, we applied a mixture of known alleles to the lanes on both sides of each gel.

Genetic differentiation among populations was examined via an analysis of molecular variance using Arlequin ver. 2.000^53^. We sampled multiple workers from each nest for the genetic analysis. Given a mother-daughter (queen-worker) structure within nests, the sampling of multiple workers from a nest may result in pseudoreplication in the statistical tests. However, both *F. yessensis* and *F. truncorum* are highly polygynous, and because workers move freely between nests^35^, ants from adjacent nests can be considered to form a homogeneous population. Indeed, previous studies using highly polydomous *Formica (s str.)* spp. have shown that relatedness is not different from zero within castes (queens or workers) in nests^37^,^54^. To confirm this, the relatedness among workers in a nest was estimated for all the populations using microsatellite data. If the average relatedness between workers in a nest is not different from zero, our sampling regime would indicate that workers’ genotypes reflect queens’ genotypes. We used RELATEDNESS ver. 5.0.8 to calculate the relatedness between workers. Furthermore, we adopted a severe significance level (p = 0.001) in this analysis to account for any concerns related to artificial decreases in p-values.

In the phylogenetic analysis, we employed the chord distance (Dc)^55^ as a measure of the genetic distance between populations because, compared with other measures of genetic distances, Dc accommodates disturbances, such as fluctuations in population size, changes in allele number due to mutations and difference in the degree of genetic differentiation between populations^55^. The Dc values were calculated using Gendist (PHYLIP, ver.3.5c)^56^. The NJ tree with by-locus bootstrap values for each node was reconstructed using the Neighbor and Seqboot programs in the PHYLIP package^56^.

### Reconstruction of ancestral character states

We used the random Ornstein-Uhlenbeck model described by Kutsukake & Innan^26^ to reconstruct the ancestral state of each character. For each reconstruction trial, we generated random values for the simulation parameters as follows: the strength of stabilizing selection (α) from a uniform distribution on the interval (0, 1), the adaptive optimum state (β) and the state of the most recent common ancestor (MRCA) from a uniform distribution on the observed interval of the character, and the base-line evolutionary rate per branch length unit from a uniform distribution on the interval (1–4,000). Then, using the parameter values, we simulated the evolution of the phenotype on the entire tree and calculated a log-likelihood value based on a comparison of the estimated and observed phenotypes. We repeated this trial for 100,000 parameter sets and selected the best 100 trials that showed higher likelihoods than the other trials. The ancestral state of each branching point was estimated as the average of these 100 results. The amount of change along a phylogenetic branch was calculated for all branches, and its correlation to branch length was examined for each character. In addition, the evolutionary correlation among the characters used were then examined using the independent contrast method^24^ to control for effects of phylogenetic structure^57^,^58^.

## Additional Information

**How to cite this article**: Imai, S. *et al*. Difference in evolutionary patterns of strongly or weakly selected characters among ant populations.. *Sci. Rep.* **6**, 39451; doi: 10.1038/srep39451 (2016).

**Publisher's note:** Springer Nature remains neutral with regard to jurisdictional claims in published maps and institutional affiliations.

## Supplementary Material

Supplementary Information

## Figures and Tables

**Table 1:** Residual variance of each character in each population.

	Populations								
	Kawayu	Obihiro	Furano	Moshiri	Ishikari	Ohnuma	Gotenba	Norikura	Average ± S.D.
FTSL	20.8	30.1	13.3	18.5	21.3	10.9	9.35	12.0	17.0 ± 6.98
HTL	7.7	6.8	7.3	6.5	9.8	6.2	5.3	5.8	6.9 ± 1.40
HTSL	105.7	121.4	29.8	32.7	143.0	99.8	50.40	59.7	80.3 ± 42.73

The residuals were calculated using the regression line of each character to HW. In all the populations, residual variance was significantly diverse among populations (F-test, all significant aeter Bonferroni correction) being the largest in HTSL, the intermediate in FTSL and the smallest in HTL.
