# Supplementary material for: Difference in evolutionary patterns of strongly or weakly selected characters among ant populations
Source: Sci Rep. 2016 Dec 20;6:39451. doi: 10.1038/srep39451 (PMC5171879; doi:10.1038/srep39451)

Title: Difference in evolutionary patterns of strongly or weakly selected characters among ant populations.

Authors: Shuichiro Imai, Kazuya Kobayashi, Yusaku Ohkubo, Norihiro Yagi and Eisuke Hasegawa*

Authors affiliation:

Laboratory of Animal Ecology, Department of Ecology and Systematics,

Graduate School of Agriculture, Hokkaido University.

Kita-ku, Sapporo 060-8589, Japan.

Corresponding author:

Eisuke Hasegawa

Supplementary Information

Supplementary Table S1. The slopes of the allometric equation (with 95% confidential interval) of each character on HW in each population. For each character, the variance in the slopes among the populations was tested using ANCOVA. The right column shows the results of ANCOVA.

									Average variance in slopes among populations	Results of ANCOVA			
Population	Kawayu	Obihiro	Furano	Moshiri	Ishikari	Ohnuma	Gotenba	Norikura		SS	df	*F*-value	p value
FTSL	0.909 (±0.043)	0.836 (±0.046)	0.860 (±0.033)	0.777 (±0.045)	0.891 (±0.040)	0.882 (±0.031)	0.902 (±0.027)	0.877 (±0.038)	$1.86x10^{-5}$	0.065	7	5.523	p<0.0001
HTL	1.07 (±0.026)	1.012 (±0.022)	1.003 (±0.024)	0.981 (±0.020)	1.028 (±0.027)	0.997 (±0.023)	1.012 (±0.020)	1.055 (±0.027)	$0.90x10^{-5}$	0.028	7	5.872	P<0.0001
HTSL	1.07 (±0.096)	1.033 (±0.093)	0.905 (±0.049)	0.842 (±0.047)	1.038 (±0.104)	1.176 (±0.093)	0.928 (±0.062)	1.026 (±0.085)	$11.17x10^{-5}$	0.039	7	6.917	p<0.0001

Supplementary Table S2. The results of F tests for the residual variances among FTSL, MTSL and HTSL of 50 workers collected from Ishikari population. The upper rights are F values, and the lower lefts are p values. After the Bonferroni correction (the significance level is p=0.0167) for multiple comparisons, the residual variance in FTSL is significantly smaller than that in MTSL and HTSL. Whereas, the residual variance in MTSL is not different from that in HTSL. We collected 50 workers of *F. yessensis* from a mound at Ishikari shore. For each worker, we removed head and 6 legs from the body and stuck these on a slide grass. These body parts were photographed with a scale (1mm) and were measured for head width (HW), FTSL, MTSL and HTSL by using a software (ImageJ, ver. 1. 5. 1F). Each value was log transformed and the allometric equation to HW was calculated for each of the other three traits. We then calculated the residual from the allometric regression line for FTSL, MTSL and HTSL for each individual. The difference in the residual variance was compared by F test for each pair of the three traits. We made a Bonferroni correction for multiple comparisons to judge statistical significance of each comparison.

	FTSL	MTSL	HTSL
FTSL	-	2.00809	2038975
MTSL	0.00868*	-	1.19007
HTSL	0.00155*	0.27449	-

*: Significant after Bonferroni correction.

Supplementary Figure S1. **A map of the collection sites.** Species collected and the latitudes at each site are also shown. Black squares indicate *Formica truncorum* and white square show *F. yessensis*. The map has been made from a copyright free map from http://www.freemap.jp/itemFreeDlPage.php?b=asia&s=kouiki_east (asia_kouiki_east_1.gif).

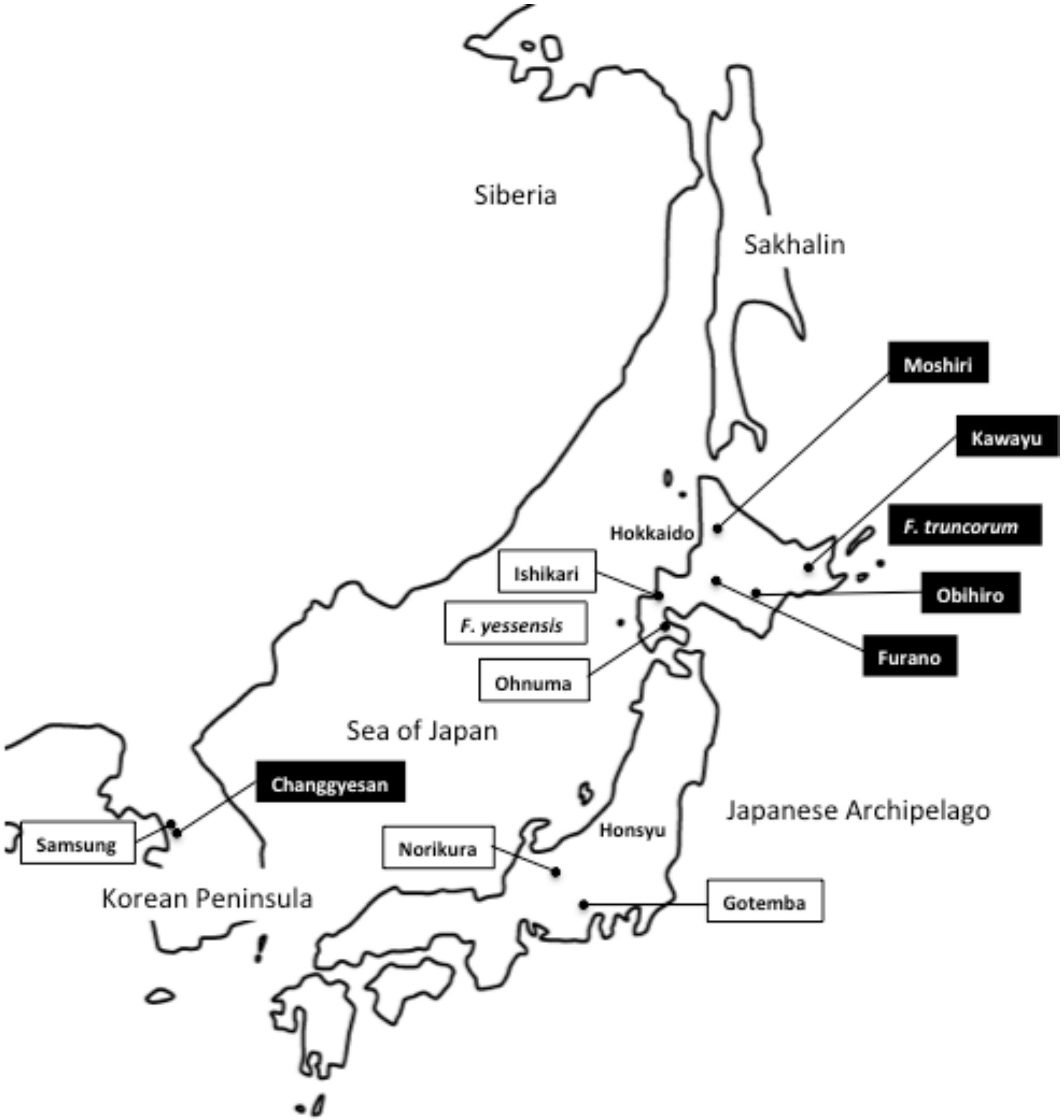

Supplementary Figure S2. **Measured characters and its landmark points for measuring.**

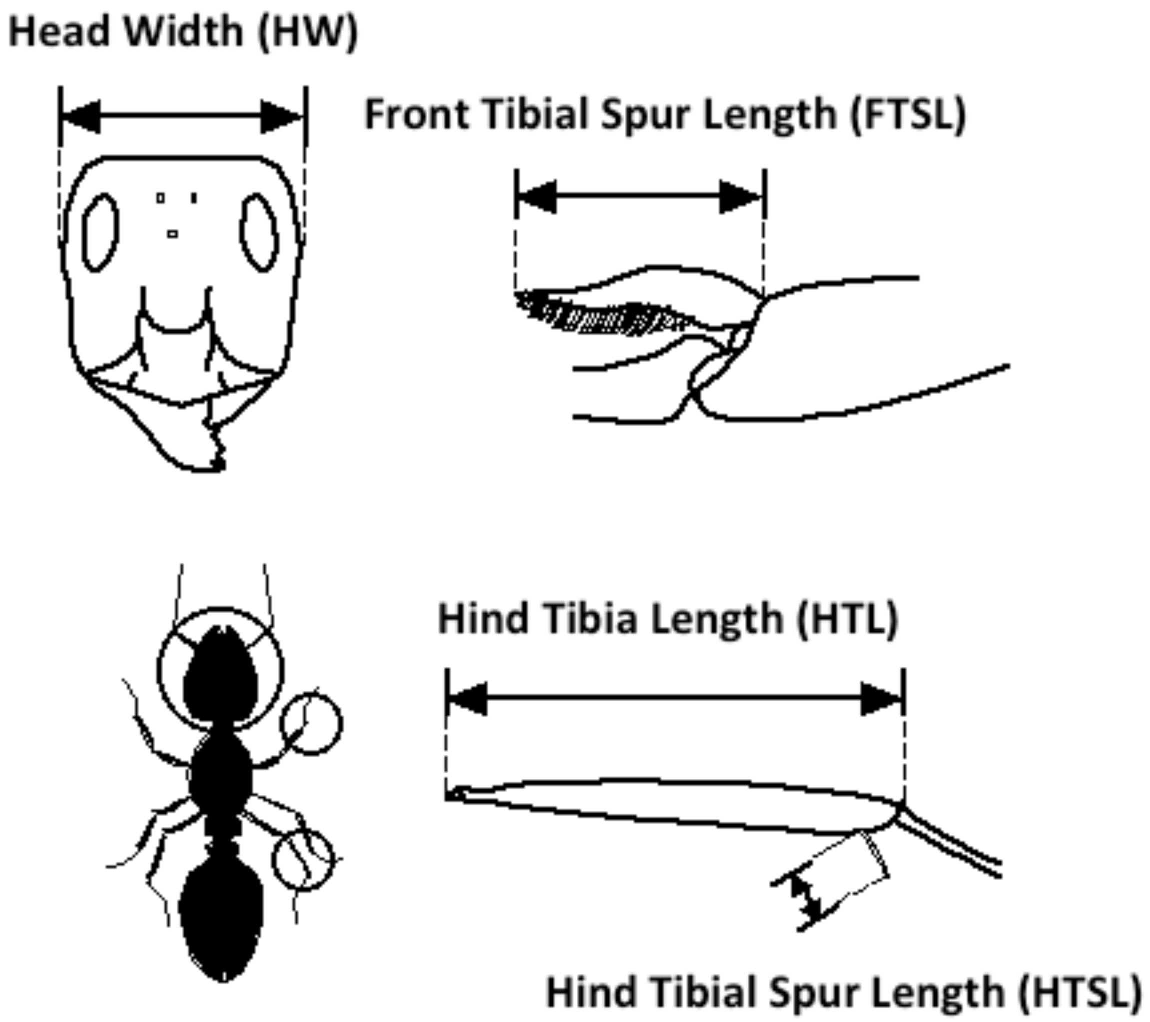

Supplementary Figure S3. Difference in walking speed of workers before and after cutting off the tibia of the right hind leg. The mean with S. D. of 50 workers from the Ishikari population are presented. Each walking worker was recorded using a digital video camera (HC-V720M, Panasonic, Osaka, Japan) during 1 minute in a plastic box (29 x 22 x 5 cm, placed by a section of paper used as a scale), the tibia of right hind leg was then cut at its midpoint. The worker was allowed to recover for 5 min in the box covered by a plastic petri dish (5.6 cm in diameter, 1.0 cm in depth). Then, the petri dish was removed, and the movement of the worker was recorded for 1 min. For each recording, we selected a period of at least 2 sec during which the worker continuously walked. The trajectory of the movement was traced using the motion tracing function of After Effect ver. 13.7.2.3 (Adobe), and total time spent moving was recorded. We took a digital photo of the trajectory using the screen-shot function of the computer used in the analysis (Power Book Pro, Apple), and the length of the trajectory was measured with ImageJ ver. 1. 5. 1F. The walking speeds of the workers were calculated from these data. We measured the walking speeds of 50 workers and compared the speeds before and after cutting off the tibia using a paired *t*-test.

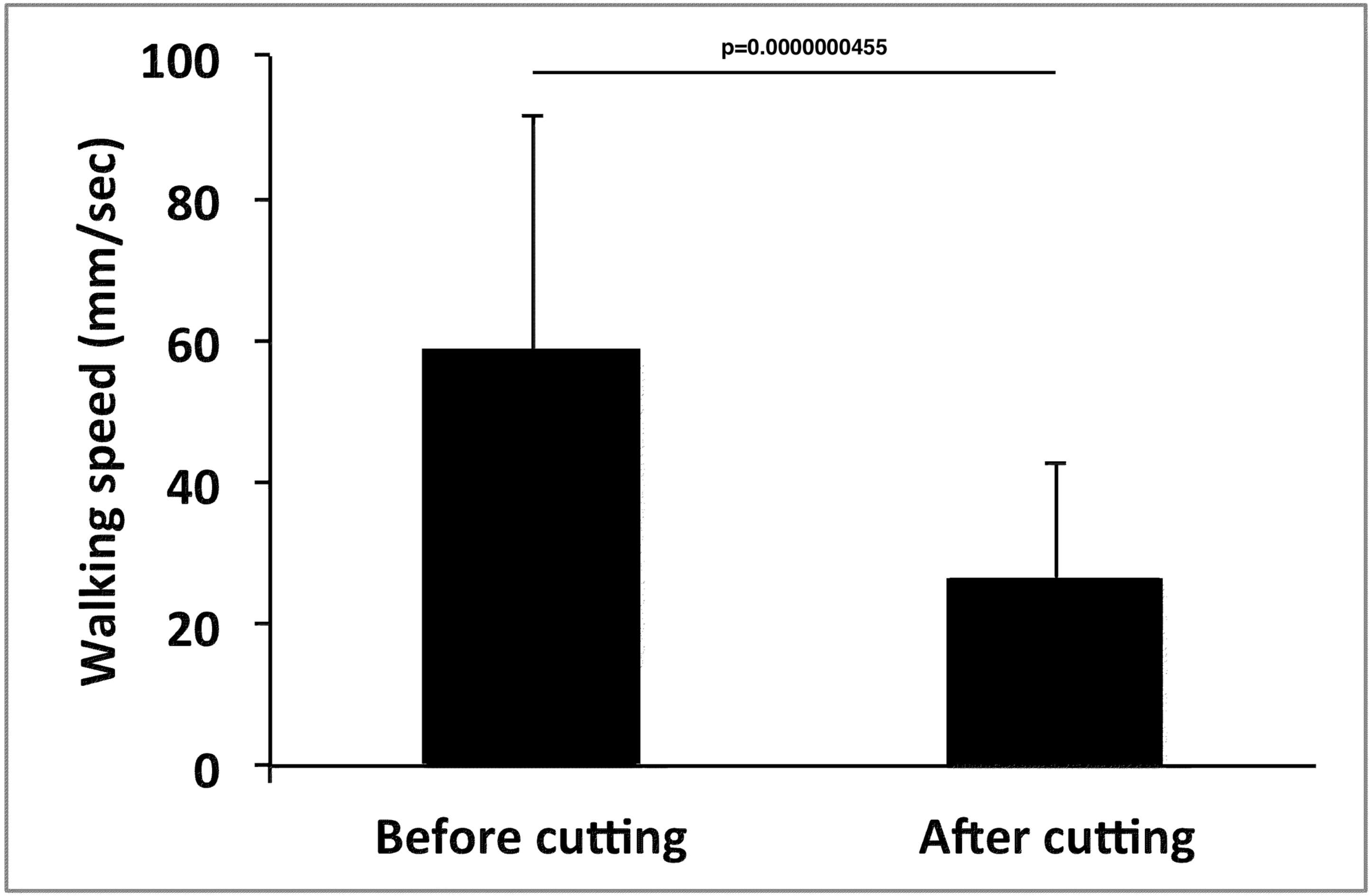

Supplement: Supplementary Information [file srep39451-s1.pdf]
